# Exploration and validation of the Ki67, Her-2, and mutant P53 protein-based risk model, nomogram and lymph node metastasis model for predicting colorectal cancer progression and prognosis

**DOI:** 10.3389/fonc.2023.1236441

**Published:** 2023-11-23

**Authors:** Chaofeng Yuan, Jiannan Huang, Yizhuo Wang, Huijie Xiao

**Affiliations:** ^1^ Department of Gastrointestinal Colorectal Surgery, The Third Bethune Hospital of Jilin University, Changchun, China; ^2^ Department of Cancer Center, The First Bethune Hospital of Jilin University, Changchun, China

**Keywords:** colorectal cancer, Her-2, Ki67, MutP53, immunohistochemistry, tumor marker, prognosis, machine-learning

## Abstract

**Introductions:**

Identifying biological markers of colorectal cancer (CRC) development and prognosis and exploring the intrinsic connection between these molecular markers and CRC progression is underway. However, a single molecular tumor marker is often difficult to assess and predict the progression and prognosis of CRC. Consequently, a combination of tumor-related markers is much needed. Ki67, Her-2, and mutant P53 (MutP53) proteins play pivotal roles in CRC occurrence, progression and prognosis.

**Methods:**

Based on the expressions by immunochemistry, we developed a risk model, nomogram and lymph node metastasis model by R software and Pythons to explore the value of these proteins in predicting CRC progression, prognosis, and examined the relationship of these proteins with the CRC clinicopathological features from 755 (training set) and 211 CRC (validation set) patients collected from the hospital.

**Results:**

We found that Ki67 expression was significantly correlated with T-stage, N-stage, TNM-stage, vascular invasion, organization differentiation, and adenoma carcinogenesis. Moreover, Her-2 expression was significantly correlated with T-stage, N-stage, TNM-stage, vascular and nerve invasion, pMMR/dMMR, signet ring cell carcinoma, and organization differentiation. MutP53 expression was significantly correlated with T-stage, N-stage, TNM-stage, vascular and nerve invasion, adenoma carcinogenesis, signet ring cell carcinoma, organization differentiation, and pMMR/dMMR. Increased expression of each of the protein indicated a poor prognosis. The established risk model based on the three key proteins showed high predictive value for determining the pathological characteristics and prognosis of CRC and was an independent influencer for prognosis. The nomogram prediction model, which was based on the risk model, after sufficient evaluation, showed more premium clinical value for predicting prognosis. Independent cohort of 211 CRC patients screened from the hospital verified the strong predictive efficacy of these models. The utilization of the XGBoost algorithm in a lymph node metastasis model, which incorporates three crucial proteins, demonstrated a robust predictive capacity for lymph node metastasis.

**Discussion:**

The risk model, nomogram and lymph node metastasis model have all provided valuable insights into the involvement of these three key proteins in the progression and prognosis of CRC. Our study provides a theoretical basis for further screening of effective models that utilize biological markers of CRC.

## Introduction

Colorectal cancer (CRC) is a common malignant tumor of the lower gastrointestinal tract. Currently, CRC has the third highest incidence of all malignancies worldwide, and it is a major risk factor for tumor-related death (1). The occurrence and progression of CRC are influenced by genetic, environmental, and lifestyle factors, such as smoking, obesity, lack of physical exercise, and alcohol abuse, as well as poor dietary habits, including a high-protein diet, a high-fat diet, excessive red meat intake, and low fruit and vegetable consumption (2, 3). These external factors act as a catalyst for CRC development, increasing the burden on economic and health resources.

The occurrence and progression of CRC have garnered significant attention from clinicians. Accurately reflecting and predicting the progression and prognosis of CRC can enhance survival rates and decrease mortality. However, current clinical practice lacks appropriate tumor markers with high sensitivity and specificity to comprehensively assess and predict CRC progression and prognosis. Therefore, it is crucial to identify relevant tumor markers with high predictive efficacy that are closely associated with various pathological features and prognosis of CRC.

Recent studies have demonstrated the significant roles of MKI67, ErbB2, and TP53 genes in the occurrence and progression of CRC, making them hot topics of research. Moreover, immunohistochemical detection of Ki-67, Her-2, and P53 proteins in pathological tissues is a common practice in daily clinical work. Thus, from a practical standpoint, detecting these three proteins can provide valuable insights into CRC progression and prognosis. However, the occurrence and progression of CRC are complex and multifactorial, involving a number of genes and signaling pathways. Moreover, there is obvious tumor heterogeneity and individualized differences among patients with CRC (4). Therefore, it is often difficult to comprehensively assess and predict the progression and prognosis of CRC based on a single molecular tumor marker. As a result, a combination of tumor-related markers is needed to more comprehensively evaluate and predict CRC progression and prognosis (5). This approach could help to provide more personalized treatment plans and reduce the morbidity and mortality rates of CRC. In line with these principles, we aimed to integrate Ki67, Her-2, and MutP53 proteins to create clinical models that can more accurately predict the progression and prognosis of CRC.

The *HER2* proto-oncogene encodes human epidermal growth factor receptor-2 (Her-2), also known as ErbB-2. Various studies have shown that Her-2 is closely associated with the progression and prognosis of certain malignancies, such as breast cancer (6) and gastric cancer (7). *HER2 (ERBB2)* gene amplification leads to Her-2 protein overexpression, which inhibits tumor apoptosis and promotes tumor cell invasion, vascular invasion, and lymphatic metastasis (8). With the progressive exploration of this gene, researchers have found that the Her-2 protein is closely related to CRC progression (9). *TP53* is an important tumor suppressor gene that inhibits the development and progression of malignant tumors. Under normal conditions, the wild-type P53 protein encoded by *TP53* inhibits cell growth and promotes apoptosis. However, *TP53* is highly susceptible to mutations, and P53 proteins translated from mutated *TP53* loses its function of regulating cell growth and apoptosis, thus promoting cancer cell proliferation (10). Ki67, a proliferating cell-associated antigen, is an indicator of the proliferative capacity of cells, and it is encoded by *MKI67*. The higher the expression of Ki67, the stronger the proliferative ability of cancer cells (11). The above evidence suggests that Ki67, Her-2, and mutant P53 (MutP53) proteins are closely related to the occurrence, progression, and prognosis of CRC.

In this study, we aimed to identify a method to more comprehensively assess the progression and prognosis of CRC based on Ki67, Her-2, and MutP53 proteins. We developed a risk model based on the expression of these proteins, as well as a nomogram prediction model based on the risk model, to evaluate the prognosis of patients with CRC. Additionally, we utilized the XGBoost algorithm to construct a model for lymph node metastasis, enabling us to accurately assess and predict the risk of CRC lymph node metastasis. The results and verifications provide new ideas for the evaluation and prediction of CRC progression and prognosis.

## Materials and methods

### Selection of clinical patients

To ensure the accuracy of the experiment and reduce the interference of confounding factors, we altogether collected the data of 755 patients who underwent standard radical surgery for CRC from August 2015 to September 2018 at The Third Bethune Hospital of Jilin University by setting a series of strict inclusion and exclusion criteria. There were 443 male patients and 312 female patients. The mean age of the male patients was 62.8 ± 10.7 years and of the female patients was 63.1 ± 10.8 years.

The inclusion criterion were as follows: 1) Patients diagnosed with CRC by cytology or pathology before surgery. 2) No anti-tumor treatment after CRC diagnosis; 3) Karnofsky Performance Status Scale score of >60. 4) Stable vital signs and consciousness. The exclusion criterion were as follows: 1) Patients with malignancies other than CRC. 2) Patients with distant metastases of CRC and who were difficult to treat with radical surgery. 3) Patients with familial adenomatous polyposis. 4) Patients taking immunosuppressive or immune-enhancing agents. 5) Patients with severe hematological, autoimmune, cardiovascular, or respiratory diseases; sepsis; uncontrollable diabetes mellitus; and/or obesity. 6) Women who were pregnant or lactating. Those 755 CRC patients were classified into training set.

### Follow-up of CRC patients

This study used the follow-up system of The Third Bethune Hospital of Jilin University, with telephone call being the main follow-up method. Follow-up was mainly used to assess the survival status and survival time of the patients. Follow-up ended in May 2023, and we collected the overall survival (OS) data of 572 patients, with a loss to follow-up rate of 24.2%.

### Measurement of Ki67, Her-2, and MutP53 expression in CRC tissues and pathological observation

We collected surgically resected tumor specimens from the 755 patients with CRC. The specimens were fixed using formaldehyde, embedded with paraffin, cut into 4-μm slices, and dewaxed and washed. A proportion of the sections were processed for pathological observation using conventional hematoxylin and eosin staining. Additional sections were incubated with 3% hydrogen peroxide solution for 15 minutes and washed with distilled water. Citric acid repair solution was added, and the sections were heated in a microwave for 10–15 minutes to repair the antigen. After cooling to room temperature, the sections were washed three times with phosphate-buffered saline (PBS). Subsequently, goat serum blocking solution was added and incubated for 15 minutes, and primary antibodies against Ki67 [Abcam Anti-Ki67 antibody (ab15580)], Her-2 [Abcam Anti-ErbB2 antibody (ab16901)], and MutP53 [Abcam Anti-P53 antibody (ab1101)] were added and incubated for 4 hours at 37°C. The wild-type P53 protein is very unstable, with a short half-life of only a few minutes, leading to its rapid degradation (10, 12). In contrast, the MutP53 proteins have significantly prolonged half-life and improved stability (13). Furthermore, before fixing the pathological specimen in formaldehyde, it is necessary to carefully dissect the specimen, locate the lesion, and check the resection range. The specimen is also presented to the patient’s family for viewing and explanation of the operation in detail, which takes approximately 20-25 minutes. Consequently, most of the wild-type P53 protein is destroyed during this period. As a result, the majority of P53 proteins detected in the immunohistochemistry are of the mutant types. Due to the TP53 gene mutations occurring at different sites, there are various types of mutant P53 proteins translated. It is important to note that the antibody used in our study does not recognize a specific type of mutant P53 protein. Therefore, in this study, the detected mutant P53 proteins were not specifically associated with any particular types. After washing with PBS, secondary antibodies were added and incubated at 37°C for 1 hour. After rinsing the secondary antibody, the color was developed with diaminobenzidine for 10 minutes and rinsed with deionized water. The slices were then re-stained with hematoxylin for 1 minute, followed by dehydration and resin sealing. The expression of Ki67, Her-2, and MutP53 proteins were measured under a microscope at 200× magnification. The immunohistochemical staining results were interpreted individually by two independent pathologists who were blinded to the patients’ clinicopathological and prognostic information, before being judged by a third pathologist. The tumor–node–metastasis (TNM) stage of CRC was determined according to the criteria outlined by the American Joint Committee on Cancer (AJCC), 8th edition.

### Determination of Ki67, Her-2, and MutP53 immunohistochemical staining results

**Figure 1:**
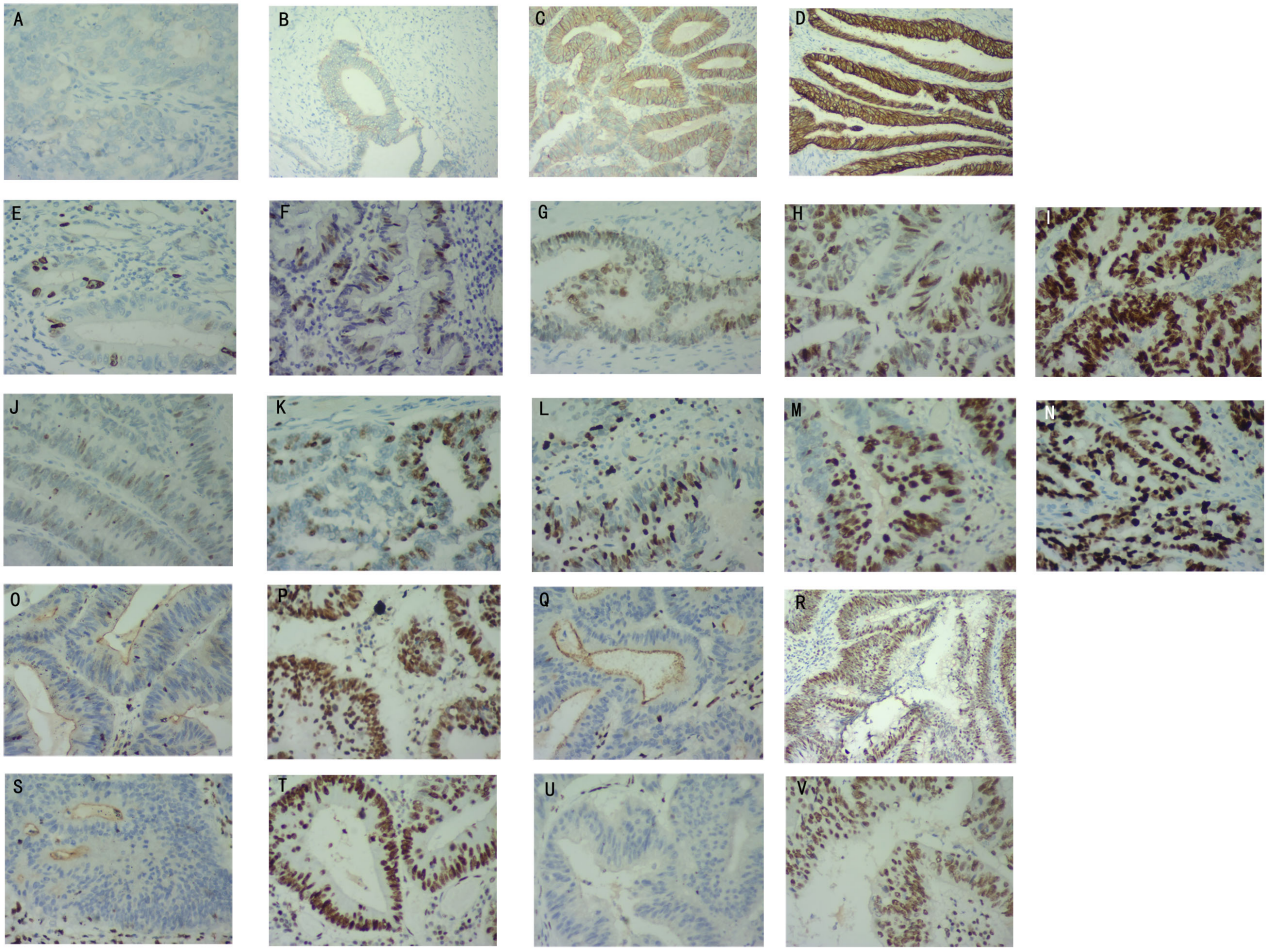
Determination of Her-2, MutP53, Ki67, and Mismatch repair (MMR) proteins immunohistochemical staining results. Her-2 protein expression levels of negative **(A)**, 1+ **(B)**, 2+ **(C)**, 3+ **(D)**. MutP53 proteins expression levels of 10%+ **(E)**, 30%+ **(F)**, 50%+ **(G)**, 70%+ **(H)**, 90%+ **(I)**. Ki67 protein expression levels of 10%+ **(J)**, 30%+ **(K)**, 50%+ **(L)**, 70%+ **(M)**, 90%+ **(N)**. MLH1 protein negative **(O)** and positive **(P)** expression. MSH2 protein negative **(Q)** and positive **(R)** expression. MSH6 protein negative **(S)** and positive **(T)** expression. PSM2 protein negative **(U)** and positive **(V)** expression.

The expression of Ki67 and MutP53 proteins were determined based on the staining depth and the percentage of cells with positive staining. Her-2 protein expression was rated based on the Valtorta scoring criteria (14). **Figures 1A-D** displays the expression levels of Her-2 proteins, categorized as negative, 1+, 2+, and 3+. The expression levels of MutP53 proteins, ranging from 10%+ to 90+, are presented in **Figures 1E-I**. Additionally, **Figures 1J-N** illustrates the expression levels of Ki67 proteins, which include 10%+, 30%+, 50%+, 70%+, and 90%+.

### Mismatch repair protein detection and dMMR/pMMR definition

The tumor specimens were fixed using formaldehyde and embedded with paraffin for sectioning. The immunohistochemical procedure is described above. Immunohistochemical staining of MMR proteins was performed using anti-MMR protein antibodies [Abcam Anti-Mismatch Repair antibody (ab92471, ab110638, ab14206, ab70270)]. Deficient mismatch repair (dMMR) was defined as one or more MMR protein expression deficiencies. MMR protein expression deficiencies were defined as the absence of nuclear staining in tumor cells, while positive nuclear staining was present in normal colonic epithelium and lymphocytes. If all four MMR proteins were expressed (any degree and proportion of tumor cell nuclear staining was determined as the presence of protein expression), the tumor was considered as proficient mismatch repair (pMMR). **Figures 1O, P** illustrate the expression of MLH1 protein in both negative and positive states. Similarly, **Figures 1Q, R** demonstrate the expression of MSH2 protein in negative and positive states. Lastly, the expression of MSH6 and PMS2 proteins in negative and positive states is shown in **Figures 1S–V**, respectively.

### Collection of independent validation cohort

We totally screened 211 patients who underwent radical colorectal cancer surgery from August 2015 to September 2018 at the Xinmin Branch of the Third Bethune Hospital of Jilin University according to the same inclusion and exclusion criterion, with follow-up ending in May 2023. There were 132 male and 79 female patients, with a mean age of (64.6 ± 10.1) years for males and (62.1 ± 10.3) years for females. The identification of immunohistochemical results and observation of postoperative tumor tissue pathology were identical to the previous methods.

### Statistical analysis and data visualization

The baseline data were calculated using the chi-square test. Spearman’s correlation was used to examine the relationship between the expression of the three key proteins. The correlation chord diagram was used to present the results of the correlation tests, and visualization was performed using the igraph package. The Complexheatmap package in R was used to generate the heat map, while the ggplot2 package was utilized to create the frequency histograms. The relationships between various pathological features and protein expression were examined using Wilcoxon’s rank-sum test and the Kruskal–Wallis test, and the results were visualized using the ggplot2 package. A binary logistic model was constructed using the glm function. Subsequently, the net benefit was calculated using the rmda package. Lastly, the DCA results were visualized using the ggplot2 package.

The survival analysis of patients with CRC with different pathological features and different levels of protein expression was performed using the Survival package, and Kaplan–Meier curves were drawn using the survminer package of R software. We used the STRINGdb package to analyze proteins in the STRING database (15) that are closely linked to Ki67, Her-2, and P53 proteins and to depict the interaction network. We then developed a risk model using the multivariate Cox regression analysis based on Ki67, Her-2, and MutP53 proteins expression, as well as the survival data of the 572 patients with CRC. The risk score was calculated by 0.0337 × (Ki67) + 0.4826 × (Her-2) + 0.0216 × (MutP53). We applied this equation to calculate the risk score of each of the 755 patients, which were subsequently stratified into two groups using the median risk score as the cut-off. Patients with scores higher than the median were allocated to the high-risk group, while patients with scores lower than the median were allocated to the low-risk group. The forest plot of the risk model was visualized using the ggplot2 package of R, the alluvial plot was processed using the ggalluvial package of R, the survival differences between the high- and low-risk groups were analyzed using the Survival package of R. The Kaplan-Meier curves and cumulative hazard curves were constructed using the survminer package of R. We used the timeROC and ggplot2 packages to analyze and characterize the time-dependent receiver operating characteristic (ROC) curves of the risk model and the expression of the three proteins. The risk factor maps of the risk model were visualized using the ggplot2 package of R. The ggplot2 package was used to depict the principal component analysis (PCA) graph after dimensionality reduction of the risk model using the PCA method. We also performed a calibration analysis of the risk model using the Survival and rms packages of R and depicted the prognostic calibration curve.

We analyzed the differences between the risk scores and various pathological characteristics of patients with CRC using the Wilcoxon rank-sum test and Kruskal–Wallis test, and we used the ggplot2 package of R to visualize the results of the analysis. We used the rms package of R to analyze the degree of fitting between the risk model and the different pathological features of CRC and to delineate diagnostic calibration curves. The ResourceSelection package was used to analyze the risk model calibration measures. We then chose the pROC package to analyze, compare, and depict the diagnostic ROC curves and PR curves of the risk model and the expression of the three proteins in specimens with different pathological features.

We used the univariate and multivariate Cox proportional hazards regression analysis to analyze the effects of various factors on the prognosis of patients with CRC, and we drew forest plots using the ggplot2 package of R. We used the rms package to build the nomogram prediction model and to draw the prognostic column line diagram. The rms package of R was used for prognostic calibration analysis and visualization of the nomogram prediction model. We classified 572 patients into low, medium and high hazard groups according to the tertile of the nomo score. The survival and risk differences among the three hazard groups were analyzed using the Survival package, and the Survminer package was deployed to depict the Kaplan-Meier curves, cumulative hazard curves and cumulative event curves. Then the t-SNE analysis of three hazard groups were caculated by the Rtsne package and the results were visualized using the ggplot2 package. We then employ the timeROC package to analyze and profile the time-dependent ROC curve of the nomogram prediction model. We further performed Cox regression analysis for the nomogram prediction model using the Survival package, and the ggplot2 package was implemented for delineating the prognostic proportional hazard plot. The pROC package of R software was used to analyze and depict the prognostic ROC curves for the nomo scores, risk scores, and various independent influencing factors. Next, we fit the prognostic model using the survival package and inscribed the prognostic decision curve analysis (DCA) curves using the stdca.R package. And then we utilized the rms package to implement the construction of the restricted cubic spline model and conduct correlation analysis. Additionally, we employed the plotRCS package to visualize the results. For the depiction and analysis of the error line plots of the concordance index, we leveraged the ggplot2 package. Finally, the Python Nricens1.6 package was employed to perform net reclassification index (NRI) analysis, comparing the prognostic prediction accuracy of the nomo score and risk score.

In addition, we deployed the same approach and calculation equation to establish the risk model and nomogram prediction model in the validation set. All the 211 patients were stratified into different risk groups and hazard groups according to the same criterion in the training set. The methods for analyzing and depicting the alluvial plot, survival curves, cumulative hazard curves, prognostic calibration curves, time-dependent ROC and prognostic proportional hazard plots were the same as the above procedures.

Based on the expression levels of Ki67, Her-2, and MutP53 proteins, we analyzed two cohorts comprising 755 and 211 CRC patients. Nine different machine-learning algorithms including XGBoost, LightGBM, AdaBoost, Logistics, GaussianNB, ComplementNB, Kneighbors, SVM, Multi-layer perception were employed to develop a lymph node metastasis model using resampling methods. The random seed was set to 42, with a validation group proportion of 20% and a fold of 10. These procedures were performed using Python version 3.7. The best machine-learning algorithm was selected based on ROC, PR, DCA curves, calibration plot, and forest plot. The SHAP model (SHAP package, version 0.39.0, Python 3.7) was then applied to illustrate the contribution and importance of the three key proteins in the predictive lymph node metastasis model. Finally, we randomly select three samples to provide detailed explanations on how these key proteins influence the model using force plots.

## Results

### Overall pathological characteristics of patients with CRC and the relationship between Ki67, Her-2, and MutP53 proteins expression

**Figure 2:**
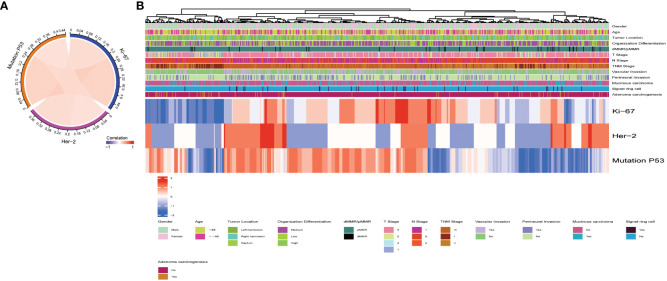
Overall pathological characteristics of patients with CRC and the relationship between Ki67, Her-2, and MutP53 expression. Relationship between Ki67, Her-2, and MutP53 protein expression **(A)**. General situation of Ki67, Her-2 and MutP53 protein expression in 755 CRC patients with different pathological characteristics **(B)**.

The baseline data for overall pathological characteristics of the 755 patients with CRC are presented in **Table 1A**. According to the presence of lymph node metastasis, we classified TNM stages I and II as early CRC and TNM stage III as advanced CRC. We employed the chi-square test to detect potential disparities between early and advanced CRC in terms of sex, age, tumor location, degree of organization differentiation, nerve invasion, vascular invasion, pMMR/dMMR, presence of mucinous carcinoma component, signet ring cell carcinoma component, and adenoma carcinogenesis. Age, sex, tumor location, mucinous carcinoma component, and adenoma carcinogenesis did not differ significantly between early CRC and advanced CRC (P > 0.05 for all). The level of organization differentiation, pMMR/dMMR, vascular invasion, nerve invasion, and signet ring cell carcinoma component were significantly different between the two groups (P < 0.05 for all). To investigate the correlation between the expression of Ki67, Her-2, and MutP53 proteins, we employed Spearman’s correlation test, and the results were visualized using a correlation chord plot (**Figure 2A**). The correlation coefficients and correlation test results between the expression of the three proteins are listed in **Table 1B** and **Table 1C**. The graph shows a positive correlation between the expression of the three proteins (P < 0.05). However, the correlation coefficient values indicate weak expression relations between these proteins. Furthermore, in order to provide a direct visualization of the overall expression patterns of Ki67, Her-2, and MutP53 proteins in 755 CRC patients with varying pathological characteristics, a heat map was created (**Figure 2B**).

**Table 1A:** Overall pathological characteristics of patients with CRC.

Characteristics	TNM stages I & II	TNM stage III	Total	P value
**n**	390	365	755	
Sex, n (%)				
Male	229 (30.3%)	214 (28.3%)	443	0.980
Female	161 (21.3%)	151 (20.0%)	312	
Age (years), n (%)				
≤65	237 (31.4%)	200 (26.5%)	437	0.097
>65	153 (20.3%)	165 (21.9%)	318	
Organization differentiation, n (%)				
High differentiation	40 (5.3%)	7 (0.9%)	47	4.02e^−24^
Medium differentiation	282 (37.4%)	169 (22.4%)	451	
Low differentiation	68 (9.0%)	189 (25%)	257	
Tumor location, n (%)				
Right hemicolon	94 (12.5%)	85 (11.3%)	179	0.771
Left hemicolon	102 (13.5%)	104 (13.8%)	206	
Rectum	194 (25.7%)	176 (23.3%)	370	
Mismatch repair (MMR), n (%)				
Proficient mismatch repair (pMMR) 359 (47.5%)	354 (46.9%)	713	0.003	
Deficient mismatch repair (dMMR) 31 (4.1%)	11 (1.5%)	42		
Vascular invasion, n (%)				
Yes	60 (7.9%)	218 (28.9%)	278	1.57e^−36^
No	330 (43.7%)	147 (19.5%)	477	
Perineural invasion, n (%)				
Yes	63 (8.3%)	143 (18.9%)	206	1.27e^−12^
No	327 (43.3%)	222 (29.4%)	549	
Mucinous carcinoma component, n (%)				
Yes	87 (11.5%)	85 (11.3%)	172	0.748
No	303 (40.1%)	280 (37.1%)	583	
Signet ring cell carcinoma component, n (%)				
Yes	6 (0.8%)	31 (4.1%)	37	9.71e^−6^
No	384 (50.9%)	334 (44.2%)	718	
Adenoma carcinogenesis, n (%)				
Yes	33 (4.4%)	23 (3.0%)	56	0.258
No	357 (47.3%)	342 (45.3%)	699	

**Table 1B:** Table of correlation coefficients.

	Ki67	Her-2	MutP53
**Ki67**	-	0.209	0.261
**Her-2**	0.209	-	0.179
**MutP53**	0.261	0.179	-

**Table 1C:** Correlation test form (P value).

	Ki67	Her-2	MutP53
**Ki67**	-	6.69e^-9^	3.3e^-13^
**Her-2**	6.69e^-9^	-	7.08e^-7^
**MutP53**	3.3e^-13^	7.08e^-7^	-

### Determination of Ki67, Her-2 and MutP53 immunohistochemical staining results

**Figure 3:**
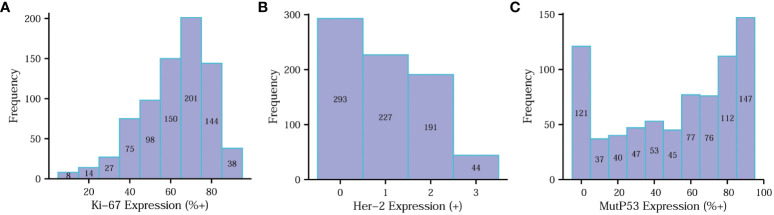
Determination of Ki67, Her-2 and MutP53 proteins immunohistochemical staining results. Determination of Ki67 **(A)**, Her-2 **(B)** and MutP53 **(C)** proteins immunohistochemical staining results.

To clearly illustrate the frequency distribution of the expression levels of the three key proteins, we constructed separate frequency histograms (**Figure 3**). In **Figure 3A**, it is evident that Ki67 70%+ expression levels exhibited the highest frequency distribution. **Figure 3B** demonstrates a gradual decrease in frequency distribution as the expression level of Her-2 increases. Additionally, **Figure 3C** reveals that the MutP53 proteins’ expression level of 90%+ accounted for the highest percentage.

### Comparisons between Ki67, Her-2, and MutP53 proteins expression and various CRC pathological features

**Figure 4:**
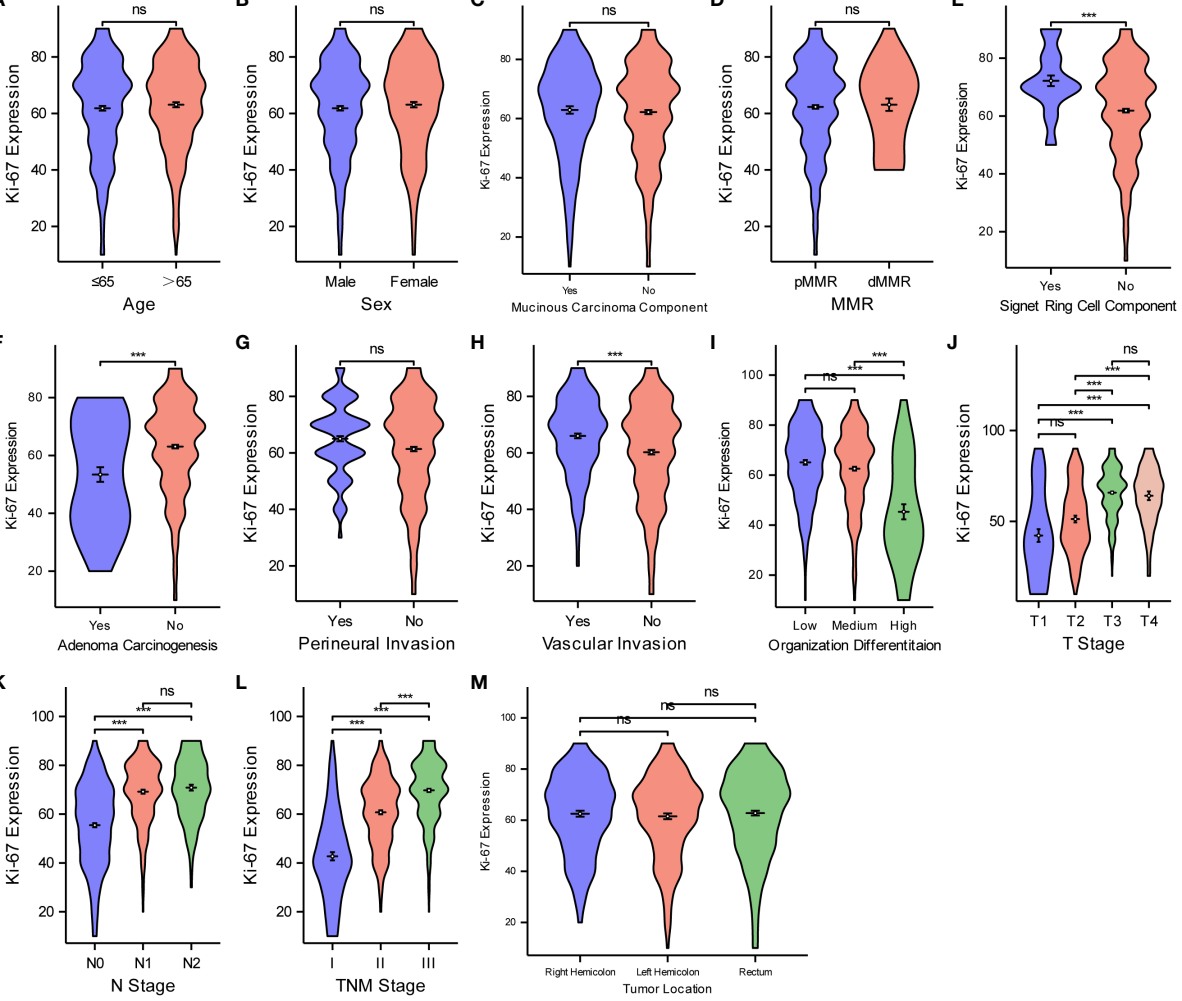
Comparisons between Ki67 protein expression and the pathological features of CRC. Comparisons between Ki67 protein expression and age **(A)**, sex **(B)**, mucinous carcinoma component **(C)**, MMR **(D)**, Signet ring cell component **(E)**, adenoma carcinogenesis **(F)**, perineural invasion **(G)**, vascular invasion **(H)**, organization differentiation **(I)**, T stage **(J)**, N stage **(K)**, TNM stage **(L)**, tumor location **(M)**. ***P < 0.001; ns, not significant.

Aiming at analyzing the relationship between Ki67 protein expression and various pathological features of CRC, the Wilcoxon rank-sum and Kruskal–Wallis tests were deployed (**Figure 4**). The violin plots suggest that Ki67 expression was not significantly different (P > 0.05) based on age (**Figure 4A**), sex (**Figure 4B**), mucinous carcinoma component (**Figure 4C**), pMMR/dMMR (**Figure 4D**), perineural invasion (**Figure 4G**), or tumor location (**Figure 4M**). Ki67 expression increased with an increase likelihood of signet ring cell carcinoma component (**Figure 4E**), degree of vascular invasion (**Figure 4H**), T-stage (**Figure 4J**), N-stage (**Figure 4K**), and TNM stage (**Figure 4L**) (P < 0.05 for all), while Ki67 expression decreased with an increase in the degree of adenoma carcinogenesis (**Figure 4F**) and organization differentiation (**Figure 4I**) (P < 0.05).

**Figure 5:**
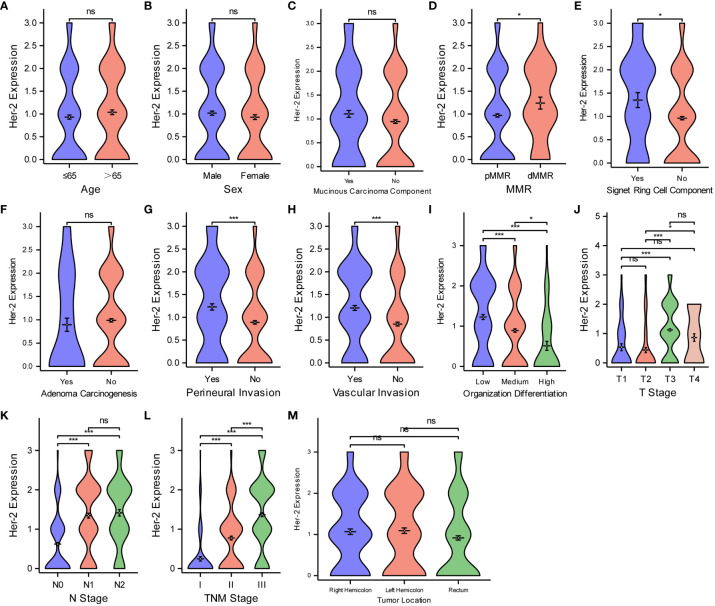
Comparisons between Her-2 protein expression and the pathological features of CRC. Comparisons between Her-2 protein expression and age **(A)**, sex **(B)**, mucinous carcinoma component **(C)**, MMR **(D)**, Signet ring cell component **(E)**, adenoma carcinogenesis **(F)**, perineural invasion **(G)**, vascular invasion **(H)**, organization differentiation **(I)**, T stage **(J)**, N stage **(K)**, TNM stage **(L)**, tumor location **(M)**. *P < 0.05; ***P < 0.001; ns, not significant.

We used the same method to analyze the relationship between Her-2 expression and the different pathological features of CRC (**Figure 5**). Her-2 expression was not associated with age (**Figure 5A**), sex (**Figure 5B**), mucinous carcinoma component (**Figure 5C**), adenoma carcinogenesis (**Figure 5F**), or tumor location (**Figure 5M**) (P > 0.05 for all). Her-2 expression gradually increased with an increase in MMR instability (**Figure 5D**), level of signet ring cell component (**Figure 5E**), degree of perineural invasion (**Figure 5G**), vascular invasion (**Figure 5H**), T-stage (**Figure 5J**), N-stage (**Figure 5K**), and TNM stage (**Figure 5L**) (P < 0.05 for all). However, as the degree of organization differentiation (**Figure 5I**) increased, the expression of Her-2 gradually decreased (P < 0.05).

**Figure 6:**
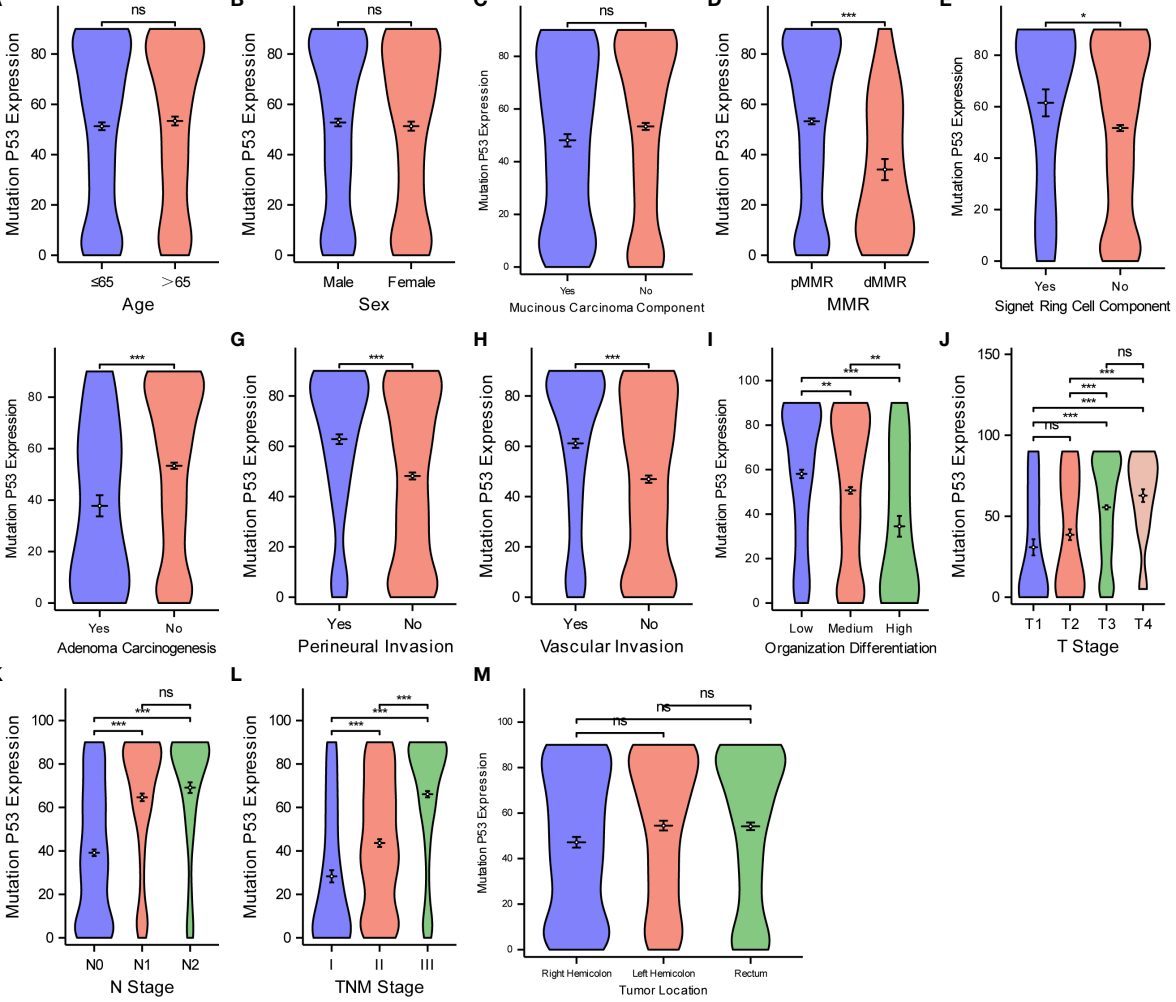
Comparisons between MutP53 proteins expression and the pathological features of CRC. Comparisons between MutP53 protein expression and age **(A)**, sex **(B)**, mucinous carcinoma component **(C)**, MMR **(D)**, Signet ring cell component **(E)**, adenoma carcinogenesis **(F)**, perineural invasion **(G)**, vascular invasion **(H)**, organization differentiation **(I)**, T stage **(J)**, N stage **(K)**, TNM stage **(L)**, tumor location **(M)**. *P < 0.05; **P < 0.01; ***P < 0.001; ns, not significant.

Finally, for the purpose of examining the expression level of MutP53 proteins in various CRC pathological features, we deploy the same analyzing methods. **Figure 6** shows that MutP53 proteins expression did not differ significantly depending on age (**Figure 6A**), sex (**Figure 6B**), mucinous carcinoma (**Figure 6C**), or tumor location (**Figure 6M**) (P > 0.05 for all). MutP53 proteins expression gradually increased with an increase in the likelihood of signet ring cell carcinoma component (**Figure 6E**), degree of perineural invasion (**Figure 6G**), and vascular invasion (**Figure 6H**); T-stage (**Figure 6J**); N-stage (**Figure 6K**); and TNM stage (**Figure 6L**) (P < 0.05 for all). However, MutP53 proteins expression decreased with an increase in the level of MMR instability (**Figure 6D**), adenoma carcinogenesis (**Figure 6F**), and organization differentiation (**Figure 6I**) (P < 0.05 for all). Overall, there was a close association between the expression of the three proteins and various pathological features in CRC.

### Comparisons of the diagnostic value of three key proteins for different CRC pathological features

**Figure 7:**
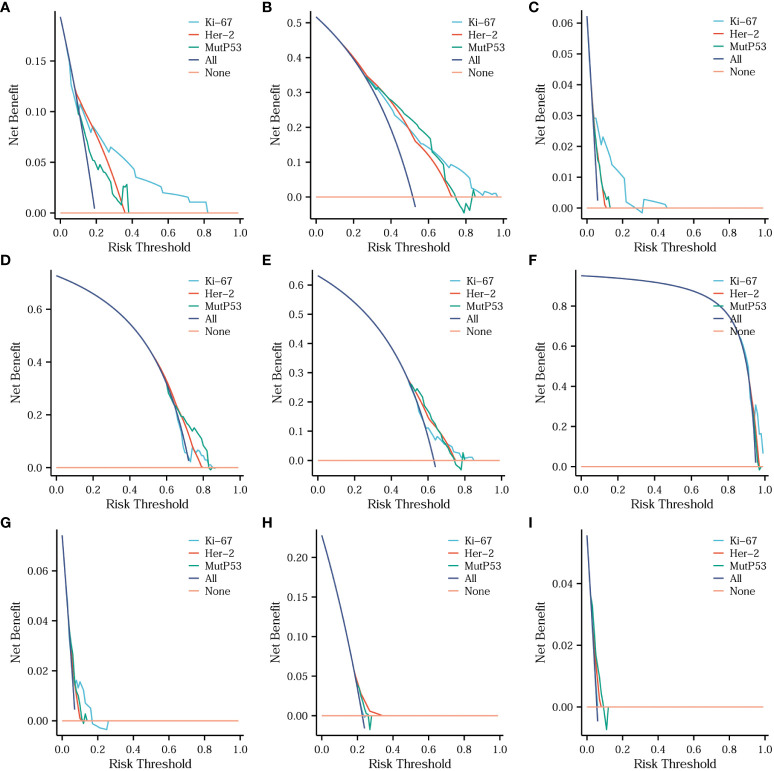
Comparisons of the diagnostic value of three key proteins for different CRC pathological features. Comparison of the diagnostic value of three key proteins for T stage **(A)**, N stage **(B)**, organization differentiation **(C)**, perineural invasion **(D)**, vascular invasion **(E)**, signet ring cell carcinoma component **(F)**, adenoma carcinogenesis **(G)**, mucinous carcinoma component **(H)** and pMMR/dMMR **(I)**.

To assess the diagnostic value of three key protein expression levels in different CRC pathological features, we employed diagnostic DCA (decision curve analysis) curves to analyze and present the comparative results. **Figure 7** illustrates that Ki67 exhibited the highest diagnostic value in determining the T stage (**Figure 7A**), N stage (**Figure 7B**), organization differentiation (**Figure 7C**), signet ring cell carcinoma component (**Figure 7F**), and adenoma carcinogenesis (**Figure 7G**). Additionally, for the diagnosis of the mucinous carcinoma component (**Figure 7H**), Her-2 displayed the highest diagnostic value. MutP53 proteins demonstrated superior performance in the diagnosis of perineural invasion (**Figure 7D**), vascular invasion (**Figure 7E**), and pMMR/dMMR (**Figure 7I**).

### Analysis of survival differences based on the expression of Ki67, Her-2, and MutP53, and various pathological features in patients with CRC

**Figure 8:**
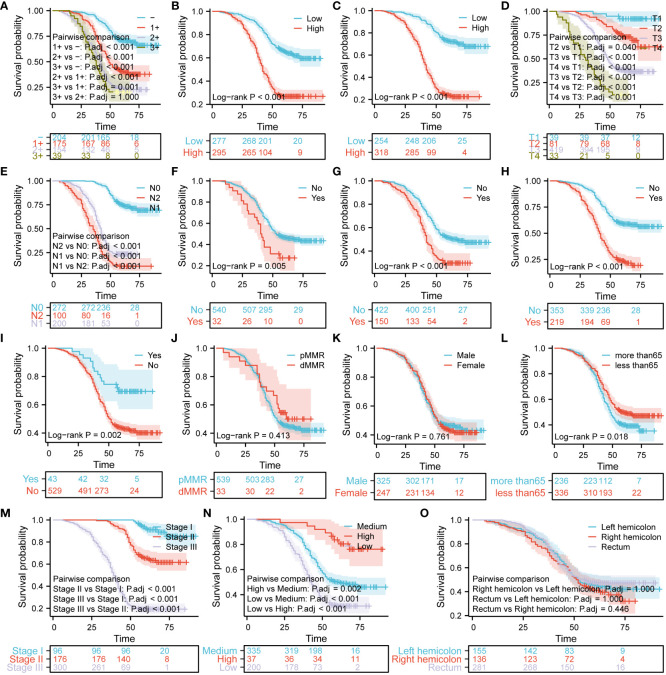
Analysis of survival differences based on the expression of Ki67, Her-2, and MutP53, and various pathological features in patients with colorectal cancer (CRC). The Kaplan–Meier curves demonstrate differences in prognosis based on Her-2 expression **(A)**, Ki67 expression **(B)**, MutP53 expression **(C)**, T-stage **(D)**, N-stage **(E)**, signet ring cell carcinoma component **(F)**, perineural invasion **(G)**, vascular invasion **(H)**, adenoma carcinogenesis **(I)**, MMR **(J)**, sex **(K)**, age **(L)**, TNM stage **(M)**, organization differentiation **(N)**, and tumor location **(O)**.

For the purpose of analyzing survival differences among 572 patients with CRC based on the expression of three proteins and different pathological characteristics, Kaplan-Meier curves were utilized. Furthermore, the log-rank test was performed to determine if there were significant variations in the survival curves. **Figure 8** shows that the higher the expression of Her-2 (**Figure 8A**), the worse the prognosis of patients with CRC (P < 0.05). Using the median Ki67 protein expression level as a threshold, we divided the patients into the low and high Ki67 expression groups. **Figure 8B** shows that the overall survival of the patients in the low Ki67 expression group was higher than that of patients in the high Ki67 expression group (P < 0.05). We used the same criteria to classify MutP53 proteins expression. **Figure 8C** shows that patients with low MutP53 proteins expression had a better overall prognosis than patients with high MutP53 proteins expression (P < 0.05). The Kaplan–Meier curves show that the overall survival of the patients gradually decreased as the T-stage (**Figure 8D**), N-stage (**Figure 8E**), level of signet ring cell carcinoma component (**Figure 8F**), perineural invasion (**Figure 8G**), vascular invasion (**Figure 8H**), and TNM stage (**Figure 8M**) increased (P < 0.05). In contrast, as the degree of adenoma carcinogenesis (**Figure 8I**) and organization differentiation (**Figure 8N**) increased, the overall survival of the patients was prolonged (P < 0.05). In addition, we found that overall survival was higher for patients aged ≤65 years (**Figure 8L**) than for patients aged >65 years (P < 0.05). In addition, the length of overall survival was not associated with pMMR/dMMR (**Figure 8J**), sex (**Figure 8K**), or tumor location (**Figure 8O**) (P > 0.05).

### Establishment and prognostic evaluation of the risk model

**Figure 9:**
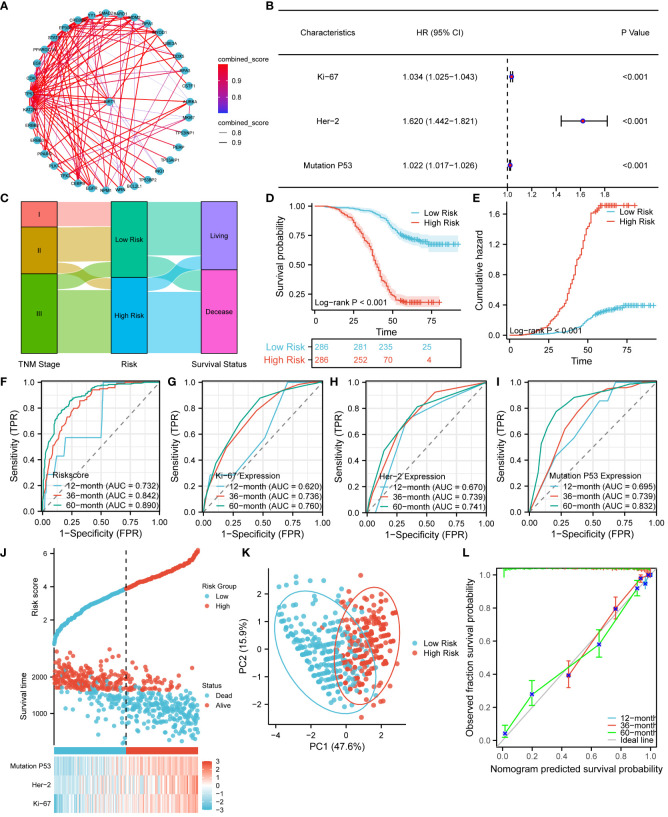
Establishment and prognostic evaluation of the risk model. **(A)** Interaction network of Ki67, Her-2, and P53 protein expression and other related proteins. **(B)** Establishment of the risk model using the multivariate Cox proportional hazards regression analysis. **(C)** Alluvial plot showing the relationship between TNM stage, risk groups, and survival status. Survival **(D)** and cumulative hazard **(E)** differences between high and low risk groups. Predictive efficacy of the risk model **(F)**, Ki67 protein expression **(G)**, Her-2 protein expression **(H)**, and MutP53 protein expression **(I)** for survival at 12, 36, and 60 months. **(J)** Risk factor plot of the risk model. **(K)** PCA plot testing the discrimination between the high-risk and low-risk groups. **(L)** Prognostic calibration curve for the risk model.

In order to explore the interactions of Ki67, Her-2, and P53 proteins within the signaling pathway, we conducted a comprehensive analysis of proteins closely associated with these three proteins in the STRING database. The resulting interaction network is presented in **Figure 9A**. Our analysis revealed a significant number of protein molecules closely linked to the three key proteins, resulting in multiple, intricate, and complex signaling pathway interactions. The intricate nature of these interactions suggests a close relationship between those three proteins. Consequently, compared to individual protein molecules, a clinical model based on the combined expression of Ki67, Her-2, and MutP53 proteins might provide a more accurate and comprehensive reflection of CRC progression and prognosis. To construct a risk model based on the expression of these proteins, we employed multivariate Cox proportional hazards regression analysis. Based on the risk model scoring equation, we assigned risk scores to 755 patients and further divided the patients into high-risk and low-risk groups according to the median score. **Figure 9B** shows the foundation of the risk model in the form of a forest plot. To vividly illustrate the correlation between the TNM stage, risk group, and survival status of the 572 patients, an alluvial plot (**Figure 9C**) was used. The figure shows that most of the patients with TNM stages I and II were in the low-risk group, while most of the patients with TNM stage III were in the high-risk group. Correspondingly, the majority of patients in the low-risk group remained alive, while the majority of patients in the high-risk group were deceased. In terms of comparing the prognosis and cumulative hazard of patients in the high- and low-risk groups, **Figures 9D, E** were delineated. Both figures clearly show that patients in the low-risk group had better overall survival and lower cumulative hazard. To assess the predictive efficacy of the risk model (**Figure 9F**), Ki67 expression (**Figure 9G**), Her-2 expression (**Figure 9H**), and MutP53 proteins expression (**Figure 9I**) on patient survival at 12, 36, and 60 months, we generated time-dependent ROC curves. The results suggest that the predictive efficacy of the risk model was the best. In order to provide a clear visualization of the correlation among the risk groups, survival status, and expression levels of the three proteins, we generated a risk factor plot (**Figure 9J**). The majority of patients in the low-risk group are still alive, and the expression of all three key proteins was lower. In contrast, the majority of patients in the high-risk group were deceased, and the expression of all three key proteins was higher than in patients in the low-risk group. To distinguish the differences among the risk groups, we employed the Principal Component Analysis (PCA) method to scale down the data and showcase the outcomes in a PCA plot (**Figure 9K**). It was not difficult to identify a discrepancy between the high- and low-risk groups. Finally, for the purpose of assessing the accuracy of the risk model for predicting patient survival at 12, 36, and 60 months, we utilized the prognostic calibration curve (**Figure 9L**). The results suggest that the risk model had a high degree of fitness.

### Relationship between the risk model and the pathological characteristics of CRC

**Figure 10:**
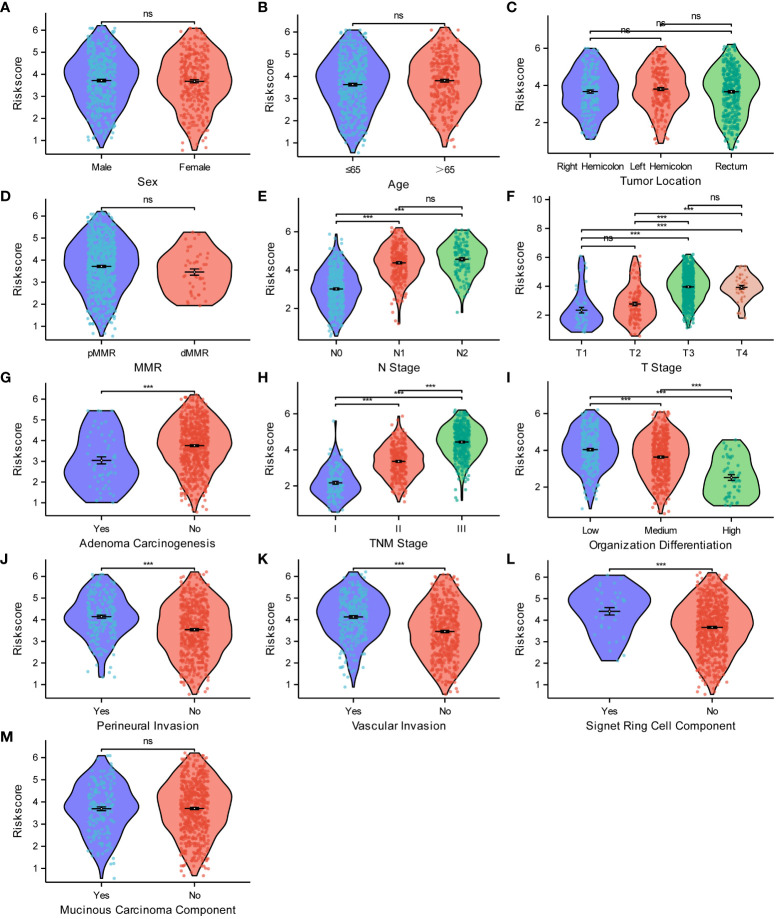
Relationships between the risk score model and the pathological characteristics of CRC. Relationships between the risk score and sex **(A)**, age **(B)**, tumor location **(C)**, MMR **(D)**, N stage **(E)**, T stage **(F)**, adenoma carcinogenesis **(G)**, TNM stage **(H)**, organization differentiation **(I)**, perineural invasion **(J)**, vascular invasion **(K)**, signet ring cell component **(L)**, mucinous carcinoma component **(M)**. ***P < 0.001; ns, not significant.

After establishing the risk model, we utilized violin scatter plots to investigate the correlation between different pathological features of CRC and the risk model. The comparative plots show that sex (**Figure 10A**), age (**Figure 10B**), tumor location (**Figure 10C**), pMMR/dMMR (**Figure 10D**), and mucinous carcinoma (**Figure 10M**) were not associated with the risk score (P > 0.05). The risk score gradually increased with an increase in N-stage (**Figure 10E**), T-stage (**Figure 10F**), TNM stage (**Figure 10H**), degree of perineural invasion (**Figure 10J**), vascular invasion (**Figure 10K**), and level of signet ring cell carcinoma component (**Figure 10L**) (P < 0.05). In contrast, the risk score gradually decreased as the degree of adenoma carcinogenesis (**Figure 10G**) and organization differentiation (**Figure 10I**) increased (P < 0.05). All of the analytic results show that the risk model was closely related to the different pathological characteristics of CRC.

### Fitness degree evaluation and diagnostic effectiveness of the risk model

**Figure 11:**
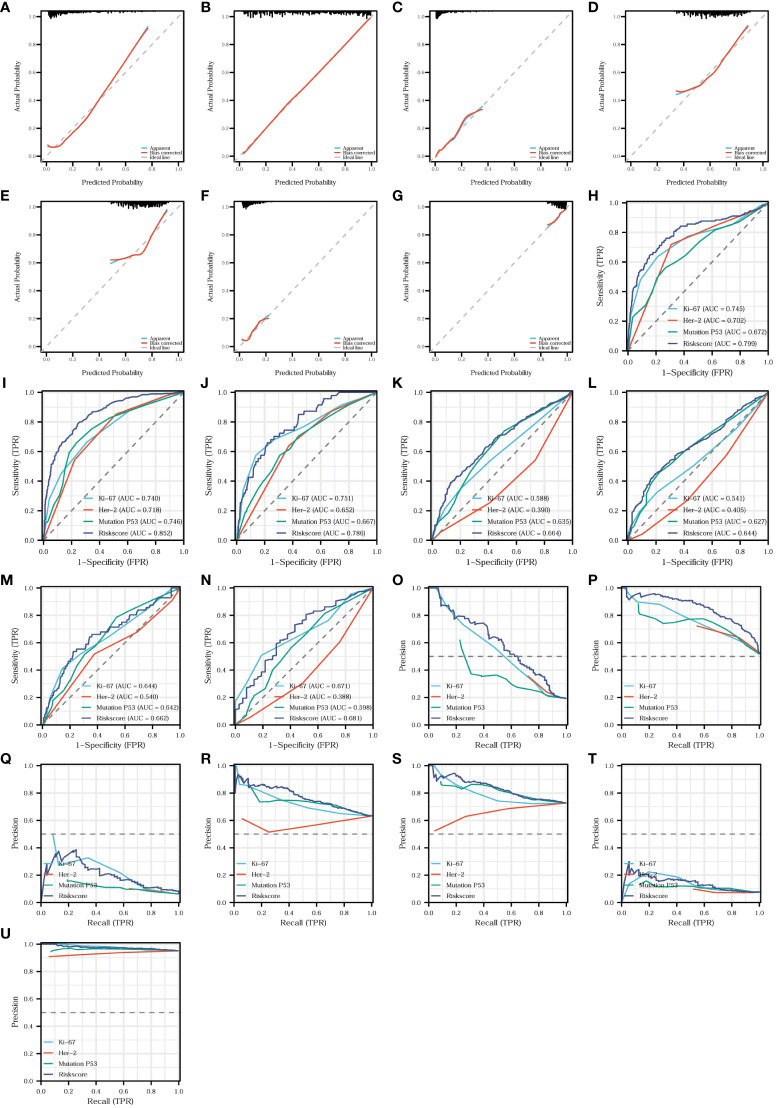
Fitness degree evaluation and diagnostic effectiveness of the risk model. Diagnostic calibration curves of the risk model for T-stage **(A)**, N-stage **(B)**, organization differentiation **(C)**, vascular invasion **(D)**, perineural invasion **(E)**, level of adenoma carcinogenesis **(F)**, and signet ring cell carcinoma component **(G)**. Diagnostic ROC curves comparing the diagnostic efficacy of the risk model and the expression of the three proteins for T-stage **(H)**, N-stage **(I)**, organization differentiation **(J)**, vascular invasion **(K)**, perineural invasion **(L)**, level of adenoma carcinogenesis **(M)**, and signet ring cell carcinoma component **(N)**. Diagnostic PR curves comparing the diagnostic value of the risk model and the expression of the three proteins for T-stage **(O)**, N-stage **(P)**, organization differentiation **(Q)**, vascular invasion **(R)**, perineural invasion **(S)**, level of adenoma carcinogenesis **(T)**, and signet ring cell carcinoma component **(U)**.

**Figure 12:**
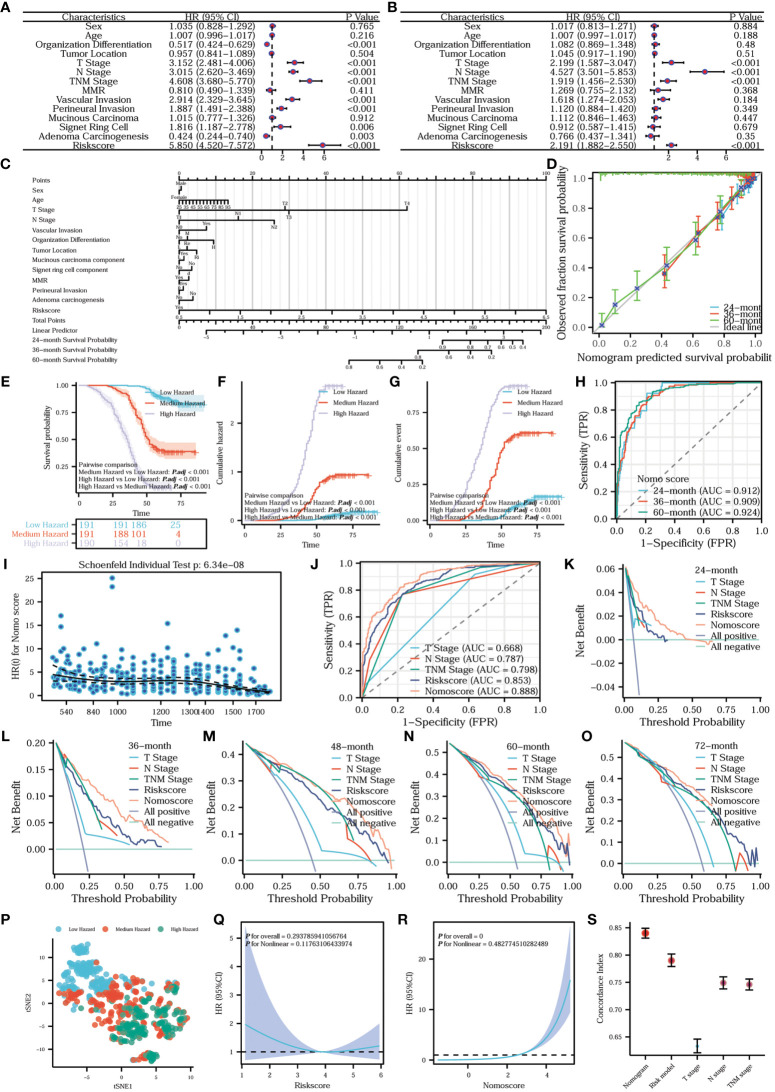
Establishment, comparison, evaluation, and analysis of the nomogram prediction model. **(A)** Univariate Cox regression forest plot and **(B)** multivariate Cox regression forest plot. The prognostic column line diagram **(C)** inscribes the nomogram prediction model. **(D)** Prognostic calibration curve of the nomogram prediction model. Survival **(E)**, cumulative hazard **(F)** and cumulative event **(G)** differences among the three hazard groups. **(H)** Time-dependent ROC of the nomogram prediction model. **(I)** Prognostic proportional risk plot of the Nomo model. **(J)** Prognostic ROC curve. Prognostic DCA curves of survival at 24 **(K)**, 36 **(L)**, 48 **(M)**, 60 **(N)**, and 72 **(O)** months. tSNE plot of nomogram **(P)**. Prognostic restricted cubic spline curves of risk model **(Q)** and nomogram **(R)**. Error line plot showed the concordance index of nomogram, risk model, T-stage, N-stage and TNM-stage **(S)**.

We found that the risk model was closely related to T-stage, N-stage, TNM stage, organization differentiation, perineural invasion, vascular invasion, level of signet ring cell carcinoma component, and adenoma carcinogenesis. Additionally, to assess the accuracy and predictive performance of this risk model for the aforementioned pathological features, we employed diagnostic calibration curves, ROC curves, and PR curves. In the diagnostic calibration curves, the diagonal dashed line represents the ideal curve, the red curve represents the calibration curve, and the blue line represents the prediction curve. The better the calibration curve fits the ideal curve, the better the model fits the actual situation and the stronger the predictive power of the model. **Figure 11** shows that the risk model has a proper degree of fitness for predicting T-stage (**Figure 11A**), N-stage (**Figure 11B**), organization differentiation (**Figure 11C**), vascular invasion (**Figure 11D**), perineural invasion (**Figure 11E**), level of adenoma carcinogenesis (**Figure 11F**), and signet ring cell carcinoma component (**Figure 11G**), especially N-stage and signet ring cell carcinoma component. We then compared the diagnostic efficacy of the risk model and the expression of the three proteins for different pathological features in **Figure 11** in the means of ROC curves and PR curves. The risk model had the highest diagnostic predictive efficacy, not only for T-stage (**Figures 11H, O**), N-stage (**Figures 12I, P**), and organization differentiation (**Figures 11J, Q**), but also for vascular invasion (**Figures 11K, R**), perineural invasion (**Figures 11L, S**), level of adenoma carcinogenesis (**Figures 11M, T**), and signet ring cell component (**Figures 11N, U**). The risk model showed a high degree of fitness and predictive diagnostic efficacy for the main pathological features of CRC.

### Establishment, comparison, evaluation and analysis of the nomogram prediction model

In order to assess the independent impact of the risk model on prognosis, we conducted univariate and multivariate Cox regression analyses, including all relevant influencing factors. The results are presented in the form of forest plots. Combining the univariate Cox regression forest plot in **Figure 12A** with the multivariate Cox regression forest plot in **Figure 12B**, T-stage, N-stage, TNM stage, and the risk model were all independent factors influencing prognosis. In addition, the hazard ratio of the risk model was >1, indicating that the risk model was a risk factor. To construct a more comprehensive clinical prediction model, we developed a nomogram prediction model that incorporates all the aforementioned influencing factors and the risk model. By utilizing this model, we obtained the nomo scores, which provide a reliable measure of risk. We inscribed the nomogram prediction model in the form of a prognostic column line plot (**Figure 12C**). To further analyze the accuracy of the nomogram prediction model in predicting survival rates at 24, 36, and 60 months, prognostic calibration curves were plotted. **Figure 12D** shows that the nomogram prediction model had a high degree of fitness for predicting the actual prognosis of the patients. All the patients were classified into three hazard groups based on the tertile of nomo scores. To compare the prognostic risk among different hazard groups, we delineated survival curves and cumulative hazard/event curves. **Figures 12E-G** vividly shows that the low-hazard group has the best prognostic outcome, the lowest cumulative risk and event. Conversely, the high-hazard group has the worst prognostic outcome, the highest cumulative risk and event. In order to distinguish the differences between the hazard groups, we utilized the tSNE method to reduce and visualize the results in a tSNE plot (**Figure 12P**). Our analysis clearly revealed a noticeable discrepancy among the high-, medium-, and low-hazard groups. What’s more, the nomogram prediction model exhibits extremely high predictive efficacy for a wide range of survival periods (**Figure 12H**). To assess whether the nomogram satisfies the proportional risk assumption, a prognostic proportional risk plot was created and analyzed. **Figure 12I** intuitively illustrates the HR values of the nomogram prediction model at different prognostic times. At the same time, the prognostic proportional risk plot indicates that the nomo score meets with the proportional risk assumption. We conducted a comparison of the predictive efficacy for prognosis using the nomo score, risk score, T-stage, N-stage, and TNM stage. To illustrate this, we generated a prognostic ROC curve (**Figure 12J**). The ROC curve demonstrates that the nomo score had the best efficacy at predicting prognosis. To assess the clinical utility of the nomo score, risk score, T-stage, N-stage, and TNM stage in predicting survival at different time points (24, 36, 48, 60, and 72 months), prognostic DCA plots were constructed. **Figures 12J–O** shows the highest clinical utility of the nomo score for patient prognosis. In order to analyze the fitting of the risk model (**Figure 12Q**) and nomogram (**Figure 12R**), the prognostic restricted cubic spline curves were examined with 3 knots. The correlations between the hazard ratio and the risk model as well as the nomogram were clearly demonstrated. To further compare the predictive accuracy and prognostic fitness of the nomo score, risk score, T-stage, N-stage, and TNM stage, the concordance index (C-index) was calculated based on the DCA curves (**Figure 12S**). The C-index values for the nomogram prediction model, risk model, T-stage, N-stage, and TNM stage were 0.840 (0.831-0.849), 0.790 (0.779-0.802), 0.633 (0.621-0.646), 0.749 (0.738-0.760), and 0.746 (0.736-0.756) respectively. The higher the C-index, the better the predictive accuracy and prognostic fitness. The results demonstrated that the nomogram prediction model had the highest predictive accuracy and prognostic fitness. To compare the prognostic prediction accuracy of the nomo score and risk score, we used the net reclassification index (NRI) analysis with an intercept value of 0.5. The nomo score was considered as the new index and the risk score as the original index. A positive NRI value indicates that the predictive accuracy of the new index is higher than that of the original one, while a negative value suggests the opposite. A value of 0 signifies no discrepancies between the two. The NRI value of 0.306 indicated that the prognostic prediction accuracy of the nomogram was 30.6% higher than the risk model. Overall, the nomogram prediction model had relatively high clinical application value.

### Validation of the risk model and nomogram prediction model

**Figure 13:**
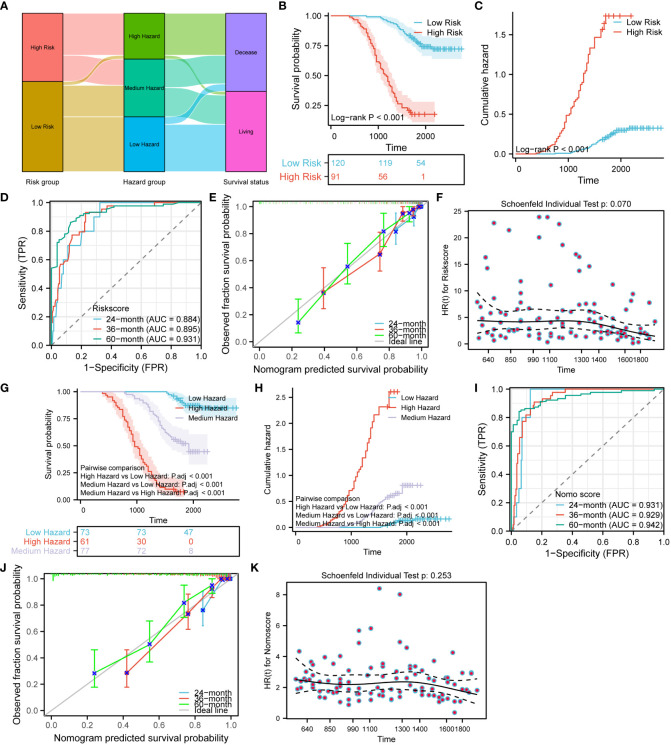
Validation of the risk model and nomogram prediction model. **(A)** Alluvial plot shows the relation between the risk groups, hazard groups and survival status. Survival **(B)** and cumulative hazard **(C)** differences between the low and high-risk groups. Time-dependent ROC **(D)**, prognostic calibration curve **(E)** and prognostic proportional risk plot **(F)** of the risk model. Survival **(G)** and cumulative hazard **(H)** differences among the three hazard groups. Time-dependent ROC **(I)**, prognostic calibration curve **(J)** and prognostic proportional risk plot **(K)** of the nomogram prediction model.

To validate the risk model and nomogram prediction model, we adopted the same modeling method and calculation equation to construct both models using the validation set consisting of 211 CRC patients. Based on the same median risk score of 3.8741 and tertile nomo score of 1.564202 and 3.404522, all the validation set patients were stratified into different risk groups and hazard groups. To examine the correlation among risk groups, hazard groups, and survival status, we employed the alluvial plot (**Figure 13A**). In order to make a direct comparison of the survival outcome among the risk groups, **Figures 13B, C** were created. The results showed that the low-risk group has better survival outcome and lower cumulative hazard. Aiming at evaluating the predictive efficacy and fitness degree, we generated time-dependent ROC curve (**Figure 13D**) and prognostic calibration curve (**Figure 13E**). Those figures vividly demonstrate that the risk model exhibits high prognostic predictive efficacy and degree of fitness. With the purpose of assessing the compliance of the risk model with the COX risk proportion assumption, we formatted **Figure 13F**. We can discern from the **Figure 13F** that the risk model in the validation set conforms to the COX risk proportion assumption and the risk score is a kind of hazardous factor. We generated **Figures 13G-J** to appraise and compare the survival outcomes, prognostic predictive efficacy, and fitness degree of the nomogram. After analyzing the survival differences among the three hazard groups, **Figures 13G, H** indicate that the survival discrepancies are significant. Furthermore, the nomogram prediction model shows stronger prognostic predictive efficacy and fitness degree (**Figures 13I, J**). Similarly, the nomogram prediction model also complies with the COX risk proportion assumption and the nomo score is a hazardous factor for CRC prognosis (**Figure 13K**).

### Establishment, evaluation, interpretation and validation of lymph node metastasis model

**Figure 14:**
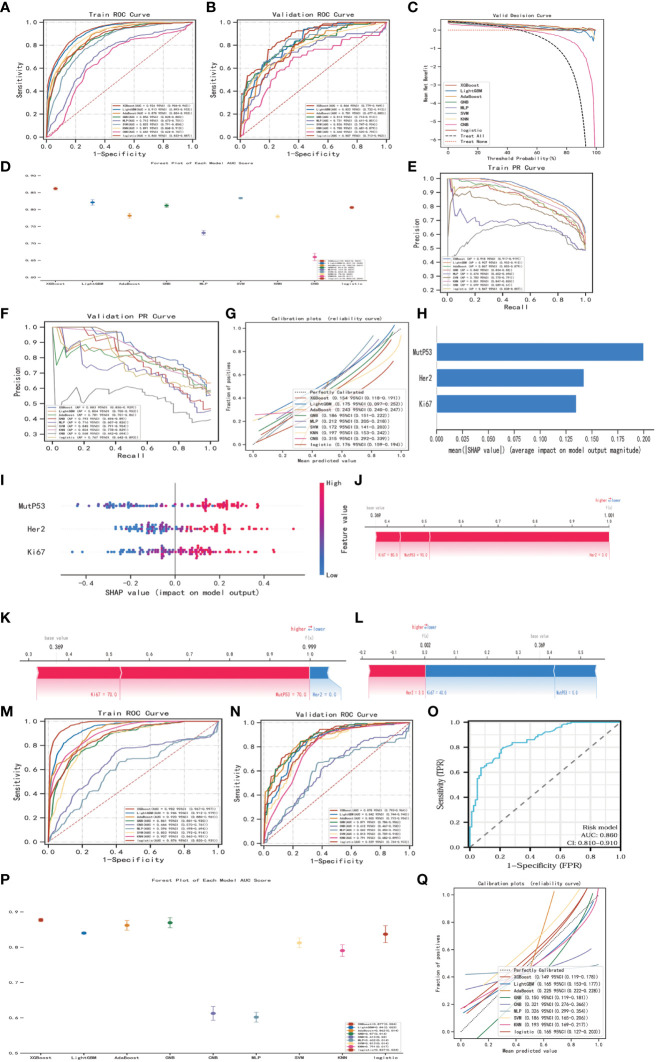
Establishment, evaluation and interpretation of lymph node metastasis model. ROC curves **(A)** and PR curves **(E)** for 9 different machine-learning algorithms of training group in training set. ROC curves **(B)**, DCA curves **(C)**, forest plots **(D)**, PR curves **(F)** and calibration plots **(G)** for 9 different machine-learning algorithms of validation group in training set. SHAP summary bar plot **(H)** and summary dot plot **(I)** of XGBoost algorithm. Force plots of three samples **(J-L)** in XGBoost lymph node metastasis model. ROC curves **(M)** for 9 different machine-learning algorithms of training group in validation set. ROC curves **(N)**, forest plots **(P)** and calibration plot **(Q)** of validation group in validation set. ROC curves **(O)** of the risk model regarding lymph node metastasis.

Aiming at establish a lymph node metastasis model based on Ki67, Her-2, and MutP53 proteins expression levels in 755 CRC patients (training set), we applied nine different machine-learning algorithms. To determine the best algorithm, we analyzed ROC, PR, DCA, calibration curves, and forest plots. From **Figures 14A, E**, it is evident that the XGBoost algorithm had the highest AUC and AP in the training group. Additionally, the XGBoost algorithm also demonstrated the highest AUC and AP in the validation group (**Figures 14B, F**). Furthermore, we plotted DCA curves (**Figure 14C**) and forest plots (**Figure 14D**). The results consistently indicated that the XGBoost algorithm possessed the strongest predictive ability. By analyzing the fitness degree of all nine algorithms using a calibration plot (**Figure 14G**), we found that the XGBoost algorithm had the lowest Brier score, signifying the highest fitness degree for predicting lymph node metastasis. In comparison to the risk model established by the multivariate COX regression method (**Figure 11I**, AUC=0.852), XGBoost proved to be the most suitable approach for the lymph node metastasis model.

In order to explain and highlight the contribution and significance of the three key proteins in the lymph node metastasis model constructed by the XGBoost algorithm, the SHAP model was utilized. **Figure 14H** clearly demonstrates that MutP53 proteins have the highest contribution in the predictive model. **Figure 14I** depicts protein expression levels, where a redder dot indicates higher expression and a bluer dot indicates lower expression. Moreover, higher SHAP values hint a greater likelihood of promoting lymph node metastasis. Therefore, the higher the expression levels of these three key proteins, the higher the probability of lymph node metastasis. Additionally, three samples were randomly selected to illustrate the lymph node metastasis model using force plots (**Figures 14J-L**). In the force plots, a blue stripe indicates a negative contribution, while a red stripe implies a positive contribution, with each stripe representing a different protein expression level. If f(x) is smaller than the basal value, the chances of tumor cells metastasizing through the lymph node decrease. On the other hand, if f(x) is larger than the basal value, the likelihood of tumor cells metastasizing through lymph nodes increases.

Finally, to further validate the lymph node metastasis model, the same approach was employed in the validation set consisting of 211 CRC patients. The XGBoost algorithm constantly exhibited superior clinical value in both the training group (**Figure 14M**) and the validation group (**Figures 14N, P, Q**), reaffirming its effectiveness. Moreover, when compared to the risk model, the XGBoost algorithm remained the most appropriate choice for the lymph node metastasis model (**Figure 14O**).

## Discussion

In this study, we systematically analyzed the differences in Ki67, Her-2, and MutP53 proteins expression in CRC in the context of various pathological features. The prognostic discrepancies between the expression of the three key proteins and various pathological features were investigated. We then established a risk model to assess the pathological characteristics and prognosis of patients with CRC and fully demonstrated the usefulness and reliability of the model. Based on the risk model, we constructed the nomogram prediction model and found that the nomogram prediction model had superior prognostic prediction efficacy and a higher degree of fitness. The risk model and nomogram prediction model provide a theoretical basis and data support for future clinical work to assess the progression and prognosis of CRC.

With the continuous development of translational medicine and molecular biology, it is being gradually recognized that the occurrence and progression of CRC are multistep and multifactorial processes. Gene mutations (16), activation or inhibition of signaling pathways (17), and alterations in the immune microenvironment (18) might affect the prognostic outcome of CRC to various degrees. In particular, gene overactivation and mutations that alter the expression of the corresponding proteins and subsequently lead to changes in a series of downstream signaling pathways are important factors influencing CRC progression. Therefore, searching for genes and proteins that play key roles in CRC progression and prognosis is indispensable in clinical research.

Ki67 is expressed in the proliferation-active G1, S, G2, and M phases of cell division, especially the G2 and M phases, while it is not expressed in the G0 phase (19). Ki67 is sensitive to proteases and is extremely easy to hydrolyze. As a result, Ki67 is not susceptible to other growth factors. Based on these features, Ki67 is closely associated with malignant tumor cells. The higher Ki67 protein expression, the stronger the proliferative ability of tumor cells and the higher the degree of malignancy (20). In a previous study, the pathological immunohistochemical results of 1800 patients with CRC showed that Ki67 protein expression was related to TNM stage and N-stage. Moreover, Ki67 expression was an independent indicator of prognosis (21). In the immunohistochemical analysis of CRC tumor tissues and paraneoplastic tissues, Ki67 expression in paraneoplastic tissues was significantly lower, and Ki67 expression gradually decreased with an increase in the distance between paraneoplastic and tumor tissues (22). All of these findings support our results. In addition, we found that the Ki67 content gradually increased as the T-stage, level of signet ring cell carcinoma component, and degree of vascular invasion increased. In contrast, as the level of adenoma carcinogenesis and organization differentiation increased, Ki67 expression decreased. Therefore, we assume that the high expression of Ki67 not only promotes infiltration and lymph node metastasis, but also promotes vascular invasion of tumor cells, which provides the basis for hematological tumor metastasis. It has also been shown that the higher the level of signet ring cell carcinoma component in CRC, the poorer the overall prognosis of the patients (23). In our present study, a high level of signet ring cell carcinoma component was strongly correlated with high Ki67 expression. We propose that Ki67 protein expression is higher in signet ring cell carcinoma, which enhances the invasive ability of signet ring cell carcinoma and is associated with poor survival outcomes. In terms of the relationship between the level of adenoma carcinogenesis and Ki67 expression, we suggest that Ki67 expression is low in benign lesions, such as adenomas, but increases after partial carcinogenesis of adenomas. When adenoma is completely cancerous and tumor tissue is filled with cancer cells, Ki67 expression will be further elevated. Consequently, elevated Ki67 expression not only promotes cell proliferation, but it may also promote cell carcinogenesis, which ultimately shortens patient survival.

*HER2*, as an important member of the HER family, is an essential proto-oncogene that is involved in the progression of several malignancies. Her-2, the tyrosine kinase receptor encoded by *HER2*, is either expressed at a low level or not expressed in healthy cells. However, when external factors lead to dimerization of Her-2 or heterodimerization of Her-2 and Her-3, the mitogen-activated protein kinase pathway and PI3K pathway are abnormally activated, which promotes frenzied cell proliferation and inhibits apoptosis (24). As a key protein that is closely associated with CRC, high expression of Her-2 promotes lymph node metastasis, increases tumor infiltration, and increases the TNM stage in CRC (25). Another study showed that the prognosis of patients with CRC with Her-2 overexpression is significantly worse than that of patients with low Her-2 expression (26). These findings support our conclusions. In addition, we found that Her-2 expression gradually increases with the degree of vascular invasion, perineural invasion, level of signet ring cell carcinoma, and MMR instability. Therefore, we believe that overexpression of Her-2 promotes vascular and perineural invasion of tumors and provides a basis for distant tumor metastasis. With regard to the observation that Her-2 expression in dMMR patients is higher than in pMMR patients, we speculate that downstream pathway alteration caused by Her-2 overexpression may lead to an expression deficiency in mismatch repair proteins, thus leading to carcinogenesis of genetically defective cells. However, this speculation still needs to be validated in further experiments. In summary, Her-2 overexpression can increase tumor malignancy by promoting infiltration, lymph node metastasis, and neurovascular invasion, increasing the level of signet ring cell carcinoma component and MMR instability, and decreasing the degree of organization differentiation, which leads to an undesirable survival outcome.

Wild type *TP53* encodes the wild type P53 protein. When cells are threatened by hypoxia, DNA damage, or radiation, amongst other factors, the wild type P53 protein accumulates and is rapidly activated, regulating a series of signaling pathway changes, leading to cell proliferation arrest, apoptosis, and senescence, thereby avoiding cell carcinogenesis (27). Therefore, wild type P53 has a significant antitumor function. However, wild type P53 is difficult to detect by immunohistochemical staining because of its short half-life and poor stability (10, 12). Mutations in the P53 protein are often derived from mutations in *TP53*. The MutP53 proteins lose their DNA binding ability and is recruited to the promoters of anti-apoptotic or proliferative genes by interacting with other transcription factors to activate the transcription of these genes, thereby promoting cancer development (28). Moreover, the half-life of the MutP53 proteins are significantly prolonged and their stability are improved (13), so it is possible to detect the MutP53 proteins by immunohistochemistry. One study showed that MutP53 protein expression is positively correlated with tumor diameter (29). Another study showed that patients with high MutP53 proteins expression have a significantly shorter survival time than patients with high expression of the wild-type P53 protein (30). Moreover, patients with CRC with dMMR have significantly lower MutP53 proteins expression than patients with pMMR (31). These findings corroborate the findings of our present study. We also found that MutP53 proteins expression was positively correlated with T-stage, N-stage, TNM stage, neurovascular invasion, and signet ring cell carcinoma component, and negatively correlated with the degree of adenoma carcinogenesis and organization differentiation. This indicates that MutP53 proteins can enhance tumor malignancy and worsen prognosis by promoting tumor infiltration, lymph node metastasis, adenoma carcinogenesis, and nerve and blood vessel invasion; increasing the level of signet ring cell carcinoma; and decreasing the degree of organization differentiation. Concerning the phenomenon that MutP53 proteins expression in patients with adenoma carcinogenesis is lower than in patients without adenoma carcinogenesis (exclusively cancerous cells in pathological sections), we regarded carcinogenesis caused by MutP53 proteins as a gradual process. With adenomas, which are benign lesions, the degree of malignancy gradually increases as the degree of carcinogenesis increases, and the MutP53 proteins are key factors in the initiation and promotion of carcinogenesis. Higher MutP53 proteins expression further weakens the cancer-suppressive effect and thus drives the cells toward malignancy. Moreover, the accumulation and activation of the MutP53 proteins may cause normal colorectal epithelial cells to develop into signet ring cell carcinoma.

It is evident that Ki67, Her-2, and MutP53 proteins influence CRC progression through different pathways. Spearman’s correlation test revealed a positive correlation between the expression of these three proteins, which is consistent with the findings of Yang et al. (32) in which Her-2 protein expression was positively correlated with Ki67 and MutP53 proteins expression. It has been elucidated that wild type P53 inhibits Ki67 expression in a dose-dependent manner and that P53 mediates transcriptional repression of Ki67 by interacting with the P53-binding motif and SP1-binding site in the Ki67 promoter, thus inhibiting excessive cell proliferation (33). In addition, it has been demonstrated that the Her-2/neu-mediated Akt pathway activates the phosphorylation of MDM2 and thus accelerates the degradation of the wild type P53 protein (34). Above all, Ki67, Her-2 and P53 proteins might be inextricably linked with each other. Further analysis of the interactions between these three key proteins through the STRING database revealed that not only are there intricate networks among the three proteins, but the three proteins also have large and complex interaction pathways with other closely related proteins. In this network, different proteins exert different effects on the tumor. There may be superimposed and containment effects among the proteins. Therefore, the effects of Ki67, Her-2, and P53 on the progression and prognosis of CRC and the interactions among them can be viewed as a complex and tangled network of pathways. Changes in the expression of a single protein not only affect tumor progression through its unique signaling pathway, but it may also alter the expression of other proteins through associated signaling pathways, which in turn influences the outcome on different levels. Therefore, investigating the effect of a single protein on tumors might have the potential to produce biased results. On this theoretical basis, we attempted to establish a risk model by applying the multivariate Cox regression method considering the expression of the three key proteins, and we comprehensively assessed the clinical value of this risk model in the pathological diagnosis and prognosis of CRC. The clinical application of a risk model based on Ki67, Her-2, and MutP53 proteins expression has not yet been reported in previous research, so we first explored the clinical usefulness and reliability of this risk model.

First, in terms of diagnosis of clinicopathological features, this risk model was closely associated with the pathological features of CRC. High risk scores were associated with higher T-stage, N-stage, and TNM stage; the level of signet ring cell carcinoma component; neurovascular invasion; a lower degree of organization differentiation; and adenoma carcinogenesis. In contrast, the risk model was independent of age, sex, and tumor location, indicating that the risk model was slightly influenced by confounding factors and highly stable in terms of the degree of tumor progression. The diagnostic efficacy of the risk model was higher than the three proteins alone, and the diagnostic predictions of the risk model for various pathological features were well fitted to the actual situation. This fully demonstrates the existence of network interactions among the three key proteins. Moreover, the value of combined measurement of the expression of the three proteins in clinical diagnostic prediction was greater than that of individual proteins. Additionally, this model may be more relevant and thus more practical and reliable for the overall assessment of patients. An increase in the risk model score represents a higher progressive stage of CRC with enhanced malignancy. Conversely, a decrease in the risk score suggests that CRC is still in the early stage and of relatively low malignancy. Therefore, during preoperative colonoscopy for CRC, we can obtain minor tumor tissue, execute immunohistochemical examination, and calculate a risk score, thus predicting the degree of tumor progression and malignancy before obtaining a postoperative pathology report.

Second, in terms of prognosis, the risk model could be used as an independent prognostic factor according to the univariate and multivariate Cox regression analyses. The risk model not only had higher prognostic predictive efficacy and prognostic clinical utility than the three individual protein indicators and the conventional T-stage, N-stage, and TNM stage, but the prognostic prediction results fit well with the actual prognosis. These observations not only demonstrate that this risk model has strong prognostic differentiation and predictive value, but it also further supports that systematic measurement of the expression of these proteins better reflects the actual tumor prognosis. An increase in the risk score predicted a poor prognostic outcome and a poor quality of life, while a low-risk score signified a relatively good prognosis. In current clinical application, carcinoembryonic antigen (CEA), as a tumor marker, occupies an important place in CRC diagnosis and treatment. However, alterations in CEA often do not reflect the actual situation. It has been noted that the variation in the CEA concentration in peripheral blood is not highly specific (35). Another commonly used tumor marker for CRC, carbohydrate antigen 19-9 (CA19-9), was less sensitive in a multicenter, controlled observation of 17,833 patients with CRC (36). It is obvious that a single index often suffers from limitations in terms of its detection ability, making it difficult to comprehensively summarize and measure CRC progression. Consequently, the establishment of joint model metrics is particularly critical in future studies. However, there are insufficient suitable model indicators to predict the pathological diagnosis and prognosis of CRC. Hence, the risk model created in this study can be further validated and improved by expanding the sample size in future work. Meanwhile, the activation or inhibition of signaling pathways, alteration of the tumor immune microenvironment, and transformation of molecular functions involved in this risk model deserve in-depth exploration. We speculate that the poor prognosis of patients with a high risk score may be attributed to the restricted function of immune cells that inhibit tumor progression in the tumor microenvironment, activation of signaling pathways that activate tumor cell proliferation and metastasis, downregulation of molecules associated with tumor apoptosis, insensitivity to inhibitory growth signals, uncontrolled cellular energy metabolism, non-mutational epigenetic reprogramming, and increased inflammatory effects of tumors. Experimental studies of these corresponding mechanistic alterations deserve further excavation and demonstration.

Based on the risk model, we continued to search for prognostic prediction models with higher clinical utility and reliability. After incorporating all relevant clinical factors, we established the nomogram prediction model. Prognostic clinical utility value analysis, prognostic diagnostic predictive efficacy analysis, and prognostic calibration analysis confirmed the superior value of the nomogram prediction model for predicting CRC prognosis. The higher the Nomo score is, the worse the prognostic outcome and higher hazard will be. We propose that the stronger efficacy of this nomogram prediction model in prognostic prediction is due to the incorporation of a complete set of factors, as well as a large prognostic sample size. In clinical practice, we can use this novel nomogram prediction model to calculate the nomo score for each postoperative CRC patient and predict their survival outcome. At the same time, the nomogram prediction model can be deployed for risk stratification of CRC patient, thus assessing the prognostic hazard more accurately. The prognostic predictive values of the risk model and nomogram prediction model are fully verified by the validation set.

Lymph node metastasis plays a crucial role in determining the prognosis of patients with CRC. Moreover, the presence and extent of lymph node metastasis also significantly impact the choice of surgical resection. Therefore, it is of utmost importance to accurately assess lymph node metastasis before surgery. Utilizing the XGBoost algorithm and considering the expression of Ki67, Her-2, and MutP53 proteins, we developed a robust and highly accurate lymph node metastasis model. This model demonstrates strong predictive ability and fitness, enabling clinicians to assess and evaluate the presence and severity of CRC lymph node metastasis effectively. Consequently, combined with imagological examination, clinicians can devise appropriate surgical interventions based on this valuable information.

The risk model, nomogram prediction model, and lymph node metastasis model have all provided valuable insights into the involvement of these three key proteins in the progression and prognosis of CRC. However, there are still limitations that need to be addressed for further improvement. One of the key areas for improvement is the expansion of clinical sample sizes in future studies. This will enhance the credibility and validity of the three models, as well as enable a more comprehensive assessment of patient survival. Moreover, it is crucial to explore the interaction mechanisms among these three key proteins and their specific roles in CRC carcinogenesis. Additionally, the immune microenvironment and drug treatment responses associated with these clinical models should be further investigated to gain a deeper understanding. By addressing these limitations, we can strive to identify more personalized therapeutic solutions for CRC patients.

## Conclusions

In general, the risk model established in this study can be used to comprehensively evaluate and predict the clinicopathological characteristics and prognosis of patients with CRC. Furthermore, we were able to discern remarkable differences in the clinical significance of CRC between the high-risk and low-risk groups, whereby the low-risk score predicted a beneficial prognostic outcome. The nomogram prediction model had higher clinical prognostic predictive efficacy. Stratifying CRC patients to different hazard groups according to the nomo scores might have a more reliable impact in clinical practice. The lymph node metastasis model established by the XGBoost algorithm might accurately assess and predict CRC lymph node metastasis, which will help clinicians develop reasonable and personalized surgical solutions for different CRC patients. Thus, the risk model, nomogram prediction model and lymph node metastasis model applied for each patient with CRC in future clinical work can comprehensively assess the progression and prognosis of CRC, which is of extraordinary clinical value for CRC diagnosis and treatment.

## Data availability statement

The original contributions presented in the study are included in the article/**Supplementary Material**. Further inquiries can be directed to the corresponding author.

## Ethics statement

The studies involving humans were approved by the ethics committee of The Third Bethune Hospital of Jilin University (No.20221020034). The studies were conducted in accordance with the local legislation and institutional requirements. The participants provided their written informed consent to participate in this study. Written informed consent was obtained from the individual(s) for the publication of any potentially identifiable images or data included in this article.

## Author contributions

Conceptualization, CY, HX and YW. Methodology, CY. Formal Analysis, CY and JH. Investigation, CY. Resources, HX. Data Curation, CY and JH. Writing – Original Draft Preparation, CY. Writing – Review & Editing, JH and HX. All authors contributed to the article and approved the submitted version.
