# Supplementary material for: Exploration and validation of the Ki67, Her-2, and mutant P53 protein-based risk model, nomogram and lymph node metastasis model for predicting colorectal cancer progression and prognosis
Source: Front Oncol. 2023 Nov 23;13:1236441. doi: 10.3389/fonc.2023.1236441 (PMC10704156; doi:10.3389/fonc.2023.1236441)
Supplement: Supplementary file 1 [file DataSheet_1.zip › Specification, Data sheets and Working sheets for all tables and figures/Figures and Tables Production Instructions.docx]

**Figures and Tables Production Instructions**

| Figures and Tables | R Package/SPSS | Data/Working sheet |
| --- | --- | --- |
| Table 1 | Chi Square Test of SPSS | Data sheet for Table 1 |
| Figure 1 and Table 2,3 | igraph Package | Data sheets for Figure 1 and Table 2,3 |
| Figure 2-4 | ggplot2 Package | Data sheets for Figure 2-4 |
| Figure 5 | Survival, Survminer Package | Data sheets for Figure 5 |
| Figure 6A | STRINGdb Package | Data and Working sheets^*^ for Figure 6A |
| Figure 6B | Survival Package | Data sheet for Figure 6B |
| Figure 6C | ggalluvial Package | Data sheet for Figure 6C |
| Figure 6D-E | Survival, Survminer Package | Data sheets for Figure 6D-E |
| Figure 6F-I | timeRoc, ggplot2 Package | Data sheets for Figure 6F-I |
| Figure 6J-K | ggplot2 Package | Data sheets for Figure 6J-K |
| Figure 6L | Survival, rms Package | Data sheet for Figure 6L |
| Figure 7  Figure 8  Figure 9  Figure 10A-B  Figure 10C  Figure 10D  Figure 10E-G  Figure 10H  Figure 10I  Figure 10J  Figure 10K-O  Figure 11A  Figure 11B-C  Figure 11D  Figure 11E  Figure 11F  Figure 11G-H  Figure 11I  Figure 11J  Figure 11K | ggplot2 Package  rms, ResourceSelection Package  pROC Package  SPSS, ggplot2 Package  rms Package  Survival, rms Package  Survival, Survminer Package  timeRoc, ggplot2 Package  Survival, ggplot2 Package  pROC Package  stdca.R Package  ggalluvial Package  Survival, Survminer Package  timeRoc, ggplot2 Package  Survival, rms Package  Survival, ggplot2 Package  Survival, Survminer Package  timeRoc, ggplot2 Package  Survival, rms Package  Survival, ggplot2 Package | Data sheets for Figure 7  Data sheets for Figure 8  Data sheets for Figure 9  Data sheets for Figure 10A-B  Data sheet for Figure 10C  Data sheet for Figure 10D  Data sheets for Figure 10E-G  Data sheet for Figure 10H  Data sheet for Figure 10I  Data sheet for Figure 10J  Data sheets for Figure 10K-O  Data sheet for Figure 11A  Data sheets for Figure 11B-C  Data sheet for Figure 11D  Data sheet for Figure 11E  Data sheet for Figure 11E  Data sheets for Figure 11G-H  Data sheet for Figure 11I  Data sheet for Figure 11I  Data sheet for Figure 11K |

* Working sheets for Figure 6A is shown below

Working sheets for Figure 6A:

Access the STRING database (https://cn.string-db.org/) Select the object for testing (Multiple proteins) List of Names (Protein to be studied)

Organisms, Homo Search Continue Exports

Export your current network As short tabular text output: download (Data sheet for Figure 6A) STRINGdb Package of R Figure 6A (Interaction network).
